# “There is hunger in my community”: a qualitative study of food security as a cyclical force in sex work in Swaziland

**DOI:** 10.1186/1471-2458-14-79

**Published:** 2014-01-25

**Authors:** Rebecca Fielding-Miller, Zandile Mnisi, Darrin Adams, Stefan Baral, Caitlin Kennedy

**Affiliations:** 1 Rollins School of Public Health, Emory University, Atlanta, USA; 2 Swaziland Ministry of Health, SNAP, Mbabane, Swaziland; 3 Futures Group, Washington, D.C., USA; 4 Johns Hopkins Bloomberg School of Public Health, Baltimore, USA

**Keywords:** HIV/AIDS, Sex work, Food insecurity

## Abstract

**Background:**

Swaziland has the highest HIV prevalence in the world – 32% of adults are currently living with HIV — and many Swazis are chronically food insecure — in 2011 one in four Swazis required food aid from the World Food Programme. In southern Africa, food insecurity has been linked to high-risk sexual behaviors, difficulty with antiretroviral therapy (ART) adherence, higher rates of mother-to-child HIV transmission, and more rapid HIV progression. Sex workers in Swaziland are a population that is most at risk of HIV. Little is known about the context and needs of sex workers in Swaziland who are living with HIV, nor how food insecurity may affect these needs.

**Methods:**

In-depth interviews were conducted with 20 female sex workers who are living with HIV in Swaziland. Interviews took place in four different regions of the country, and were designed to learn about context, experiences, and health service needs of Swazi sex workers.

**Results:**

Hunger was a major and consistent theme in our informants’ lives. Women cited their own hunger or that of their children as the impetus to begin sex work, and as a primary motivation to continue to sell sex. Informants used good nutrition and the ability to access “healthy” foods as a strategy to manage their HIV infection. Informants discussed difficulty in adhering to ART when faced with the prospect of taking pills on an empty stomach. Across interviews, discussions of CD4 counts and ART adherence intertwined with discussions of poverty, hunger and healthy foods. Some sex workers felt that they had greater trouble accessing food through social networks as result of both their HIV status and profession.

**Conclusions:**

Informants described a risk cycle of hunger, sex work, and HIV infection. The two latter drive an increased need for ‘healthy foods’ and an alienation from social networks that offer material and emotional support against hunger. Services and interventions for sex workers which address the pathways through which food insecurity generates vulnerability to HIV and social marginalization, build sex workers collective efficacy to mobilize, consider poverty alleviation, and address social and policy level changes are necessary and likely to have the greatest success.

## Background

Swaziland is a small nation in southern Africa with the world’s highest HIV prevalence — 32% of adults between the ages of 18 and 49 are currently living with the virus [1]. At national antenatal clinic sentinel surveillance sites, this number peaks at 53.8% of women aged 30–34 [2]. While HIV incidence has leveled off in recent years, the burden of disease remains high for a nation of just over one million people [3].

Two-thirds of Swazis live below the national poverty line of $1.25 per day, the unemployment rate is approximately 28%, and nearly one in four Swazis required some form of food aid from the World Food Programme in 2011 [4,5]. Inequality is widespread in the country: the top 10% of the population earns 40% of the national income, while the lowest 10% earns just 1.7% [6]. In 2011, Swaziland received a lower than expected amount of revenue from the South African Customs Union (SACU), resulting in a severe financial crisis in which escalating food prices were a point of international concern [6,7]. Legal inequality exacerbates the gendered nature of poverty in the country. As recently as 2007, sexual assault was not illegal in Swaziland, and women could not own property under customary law [8]. The government has recently enacted laws to combat gender based violence and to allow women to inherit and own property upon the death of their spouse, but these laws are only sporadically enforced [4,8]. After a prolonged lawsuit, a court hearing in July of 2013 ruled that according to Swazi law a married woman could own her own property, but that her husband was still her legal guardian and must represent her in legal proceedings [9]*.* The physical and social burdens of poverty and unemployment disproportionately affect women in the nation [4,10,11].

In southern Africa, food insecurity has been linked to an increased risk of HIV acquisition and difficulty remaining healthy for people living with HIV (PLHIV) [12-15]. Food insecurity has been linked to high risk sexual practices among women, including sex work, transactional sex, and decreased condom use [10,12,15], increased reliance on social networks for food and monetary resources [16], and decreased adherence to anti-retroviral therapy (ART) [17]. Consequently, malnutrition appears to correlate with both increased susceptibility to HIV acquisition and more rapid disease progression [12,18]. In addition to the adverse effects associated with taking the medication on an empty stomach, ART may be less effective at sustaining CD4 levels among individuals who are suffering from malnutrition or food insecurity [12]. PLHIV are frequently advised to “eat healthy foods” by health promotion campaigns and health care workers [19]. However, some studies have demonstrated that PLHIV sometimes must choose between paying for food and paying for ART [17]. Even when ART is provided free of cost by government or other health care organizations, as is the case in Swaziland, the time and money costs of transportation to the clinic may create barriers to ART adherence [14,17,20,21].

In southern Africa formal sex work and transactional sex are sometimes conflated. Both practices involve the exchange of material goods for sex, but formal sex work is characterized by self and community identification as a sex worker, whereas women who engage in transactional sex do not identify as sex workers, and their relationships tend to be socially sanctioned, or at least tolerated [22-24]. As in other countries in sub-Saharan Africa, there is some debate about the actual legal status of sex work in Swaziland, but for practical purposes it is *de facto* illegal and highly stigmatized, and women who practice street-based sex work experience frequent police harassment in the form of rape, blackmail, intimidation, and arrest [25-27]. Limited research has been conducted on the nature of sex work in Swaziland. One rapid mixed method assessment commissioned by the Ministry of Health (MoH) and the National Emergency Response Council on HIV/AIDS (NERCHA) focused on describing the HIV related knowledge, attitudes, and practices of 53 female and 8 male sex workers who were interviewed throughout the country [25]. This study found that sex workers were frequently driven by poverty and high unemployment, but that some women also saw it as a viable alternative to the legal and economic disadvantages of marriage. A quantitative study conducted with 327 female sex workers in conjunction with this project found an HIV prevalence of 60.5%, and that consistent condom use was less common with regular clients and non-paying partners as compared to new clients [28,29]. Though heterosexual sex is the main mode of transmission in Swaziland, and sex workers are considered a most at risk population both internationally and in the country [30], we could identify no studies besides the previously cited examples in either the peer-reviewed or grey literatures which focused on the needs of female sex workers who are currently living with HIV in Swaziland.

Our study objective was to examine the HIV prevention, care, and treatment needs of female sex workers who are living with HIV in Swaziland. We focused our study using a positive health, dignity and prevention (PHDP) framework. PHDP is an approach to support for PLHIV that emphasizes four main support goals: Maintaining the physical health of PLHIV, maintaining the mental health of PLHIV, preventing further transmission of HIV, and involving PLHIV in the process of research, advocacy, and care [31].

## Methods

### Setting and Participants

After consulting with key informants within the Swaziland MoH, NERCHA, the Swaziland National AIDS Program (SNAP), and NGOs which provide services to sex workers in the country, we focused our research in four different regions: A rural border town where most sex work focuses on truck drivers making their way to or from South Africa, a peri-urban community where most of the women we spoke with sold sex in bars and clubs, and two sites located in the urban Manzini-Mbabane corridor. Most sex work in the Manzini-Mbabane corridor is street-based (these locations are known as “hot-spots”), although some women also work in bars. Our inclusion criteria required that informants were over the age of 18 (the legal age of majority in Swaziland), reported that they had been previously diagnosed with HIV, and sold sex. Other research in the region has demonstrated that the line between sex work, transactional sex, and financial support within a romantic relationship can be blurry, and is largely based on the context and subjective perspective of the women involved [32-34]. To account for this, our criterion for sex work was self-identification as a sex worker. Although we had no exclusion criteria based on the last time a woman had sold sex, most participants were actively engaged in sex work at the time of data collection. Participants were recruited through support groups and peer education networks run by government public health clinics and local NGOs. NGO staff and public health nurses who worked closely with these groups were asked to refer women who were living with HIV to the study staff. If potential participants expressed interest, they were invited by the referring NGO staff member or nurse to meet the study staff at a pre-arranged interview location. Further participants were then recruited using snowball sampling methods [35].

### Community Engagement and Ethical Considerations

A community advisory committee of sex workers with representation from all four geographic areas was created to advise the study and ensure that study questions and procedures were acceptable and welcomed by the sex work community. In addition, the first author (RFM) made frequent visits to each of the four sites. These visits were used to hold meetings that explained the study to potential informants, ensure that the study was acceptable, hear potential concerns, and modify any study procedures that the community found objectionable. Meetings were conducted largely in siSwati, with occasional translations to or from English. In addition to a staff member from either the MoH or Population Services International (PSI) who had experience working with sex worker populations in the country, a member of the sponsoring NGO or public health unit whom the members trusted was always present at these meetings. RFM spoke with these group leaders before and after meetings to learn if new concerns had been raised and ensure that the study remained welcome to the community. The study received ethical approval from the Swaziland Ministry of Health’s Scientific Ethics Committee (SEC) and the Johns Hopkins Bloomberg School of Public Health institutional review board.

### Data Collection and Analysis

Two young Swazi women who were fluent in both siSwati and English conducted interviews with sex workers; RFM conducted key informant interviews and one English language sex worker interview. Interviewers were trained in qualitative research methods, ethical considerations, and the special sensitivity concerns presented by the study topic matter and informant population. Members of the community advisory committee were included on the hiring committee for these interviewers.

A total of twenty participants were recruited evenly across all four sites. Participants ranged in age from 18 to 43. The median age was 28. The final sample size was determined first by ensuring that all sample sites were equally represented, and then through iterative analyses throughout data collection that allowed the study team to reasonably conclude that data saturation had been achieved after the twentieth informant was interviewed. Two semi-structured in-depth interviews were conducted with each participant, generally with one week between interviews, in order to build rapport and revisit important themes [35]. Interview guides were designed to elicit informants’ stories about their lives as sex workers and as PLHIV. Interviews ranged from 30 to 90 minutes and were held in private offices which belonged to the local NGO or public health unit, or which had been reserved for the occasion. Verbal informed consent was obtained for each participant in siSwati or English, depending on the participant’s language preference. Participants were reimbursed for their time, equivalent to the cost of a meal and travel to the study site. All names used in this manuscript are pseudonyms.

Debriefing meetings were held between study staff and interviewers after each interview session. Larger debriefing meetings were held with the full study team each week. These meetings were used to monitor interview quality, assess themes as they emerged, use initial findings to shape later interview questions, and ensure data saturation. This iterative approach between data collection and data analysis is a hallmark of qualitative research [35].

Interviews were transcribed verbatim into siSwati, and then translated into English. The English language transcripts were then coded in Atlas.ti. Codes were generated based on the debriefing and analysis meetings that had been held throughout the course of data collection.

## Results

**Figure 1:**
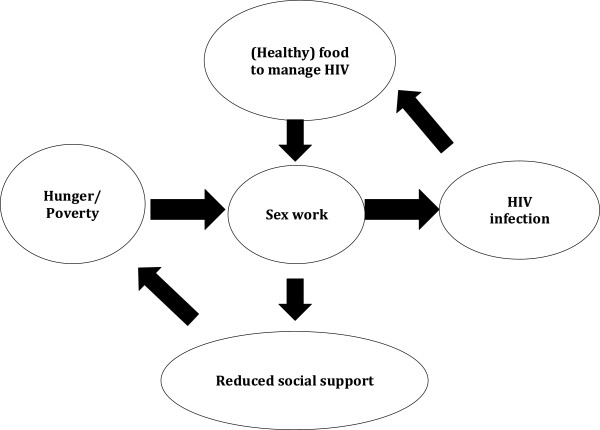
Cycle of food insecurity, sex work, and HIV.

Dual themes of hunger and poverty emerged early in the course of data collection and remained salient throughout the process. In keeping with the iterative nature of qualitative research, as the theme of hunger emerged interviewers were encouraged to probe specifically on how this fit into the lives of our informants. During analysis of the transcripts four major themes crystallized: Hunger and poverty, the relationship between sex work and HIV infection, the use of food to manage HIV status, and the effects of sex worker and PLHIV identities on social support. These were organized into a concept map (Figure 1) that shows a cyclical interaction between hunger, sex work, HIV status, and social support.

### Hunger and poverty

Informants were asked to share their stories of entry into sex work. In these narratives, hunger was frequently the initial reason for sex work entry. Entry was often linked with personal or familial poverty, and in most cases this poverty manifested as hunger. Many of our informants had children, and described initiating sex work after being left by the father of her child. In the face of high unemployment and without male support or the support of their child’s paternal family, many women described sex work as one of the few avenues available through which they could feed and support themselves, their children, and sometimes their extended families. Women described being introduced to sex work by friends, or through a slower process of individual experimentation:

What can I say; I wanted to support my child. I told myself that I was doing whatever I was doing for my child. That is how I started. Her father was not supporting her, he was denying responsibility but then said he was scared… He said a lot of things and asked me not to report the pregnancy as per Swazi custom because if his mother would know she would make him quit his studies. Ok. I agreed thinking he would own up to his responsibilities if he went to study. I thought he would come see me when he was on his holidays but he decided not to come. That is how I started since I had to provide for the child.

-Ntombifuthi (25, urban)

The father of my child was supporting [me] until we had some problems and separated… I didn’t have money to support my child and my parents were always mad at me… I started crying asking myself what I can do so that I can bring something at home… and I tried to go and hike trucks [solicit truck drivers]… The truck drivers maybe will come to me and ask “can I buy you a drink?” Then after that the person asks me, “what if we can go and have sex maybe I can give you E50” [USD 5.50]… I started to enjoy because with that fifty rand in the morning I would go and buy bread.

-Nonhlanhla (32, rural)

My friends, I would see them… going. Every sundown they would bath and go. Then one day I asked them where they were going. They told me that while I sit here, they were going to do sex working to support their children, paying [school] fees for their kids with the work they were doing.

-Nokuhanya (43, rural)

While hunger was a reason for entering sex work, few women described themselves as currently food secure. Hunger and the need to support themselves, their children, and their families were constant concerns in our informants’ lives. Many informants framed this ongoing poverty and hunger within a larger context of high unemployment and an inability to find other means of supporting themselves.

My aunt and cousin know my [HIV] status but they come to me for their plaiting [hair braiding]. However I had to supplement that money so to feed my children because I thought what if I have no customer that day what am I going to do?… There was nothing I would do really because I had to buy food for my children; for they would call to tell me food is finished.

-Phila (urban, 30)

### Food to manage HIV status

Our informants were frequently advised at local clinics that they should eat “healthy foods” to manage their HIV infection. This advice was well received, and many informants talked about “healthy foods” when asked how they coped with their HIV status. The idea of “healthy foods” was also frequently mentioned when informants were asked what services could be provided to support them. This conversation was multifaceted. Some women discussed food as a means of HIV management within the broader context of their daily poverty. For these informants, “healthy” food was equated with “any” food. In addition to situating this need within their everyday experience of poverty, some informants also made a direct link between food and their ART medications:

You don’t necessarily have to eat tasty food to take the pills. Just any food that will settle in the stomach and allow for digestion of the pills because you cannot take the pills on an empty stomach.

-Siphokazi (26, urban)

Some framed this need in the opposite way, discussing fears that poverty and an inability to access food might cause them to get sicker. This fear was frequently cited to explain an ongoing need to engage in sex work, or concerns about violence or a lack of paying customers:

Being needy is the problem because we are poor in this community… sometimes a client doesn’t pay me, and he threatens to beat you. Then I leave him be, then I don’t have anything to eat at home. Then my CD4 count drops when the person doesn’t pay me.

- Nokuhanya (43, rural)

Other women discussed the need for diet change and the integration of more “healthy” foods as a means of self-care. The term “healthy” seemed to have diverse meanings, but in general it incorporated increased fruit and vegetable consumption, decreased alcohol and fat consumption, and an increased inclusion of traditional Swazi foods into the diet (largely legumes and semi-wild greens):

“I am able to carry on with my life because they advised me on what to eat at the hospital. I eat some fruits and things like spinach, pears, peanuts, I just eat healthy food. […] I eat paw-paw [papaya] and the things I am supposed to eat. I also eat Swazi traditional food.”

-Nokubonga (31, peri-urban)

Food was an important part of HIV self-care for our informants. Diet was frequently posited as a means of gaining control over health. Many women seemed to gain a sense of comfort from this, even given the precarious nature of their economic status and the difficulty and expense that acquiring ‘healthy foods’ to comply with their medical advice created for them.

### HIV Infection

Many of our informants believed that they acquired HIV in the context of sex work. Women understood the potential hazard of HIV reinfection, and sometimes used this knowledge to negotiate condom use:

I tell them my status. One once said he had no problem with that. I just told him no! What’s mine is mine… Also what’s yours is yours, let’s not worsen each other.

-Nokulunga (28, rural)

Other informants would have preferred to use condoms to reduce their risk, but were not always able to enforce condom use. Male clients often preferred sex without condoms and were willing to pay more – or would only pay at all – for “flesh-to-flesh” sexual intercourse, as described by this informant:

Things like HIV, people come with a lot of money and tempt you. They tell you that they will pay more if you don’t use a condom. You find that the person will tempt you at the time you need money most because you have no food at home. You are putting yourself in great danger at that moment.

-Siphokazi (26, urban)

Other women thought that they had been infected through non-paying or intimate partners. Incidents of rape and coerced sex were described frequently, although only a few informants explicitly linked these stories to their own HIV acquisition:

I know how I got it, I think. I am not that sure but I think I know because I was involved with one policeman from those times when I broke up with the father of my first child. I got involved with this policeman and one time I went to his house with my friend and he said I must go and clean for him. When we were there, I found a book written “Food for people living with HIV/AIDS”. Why he was living with this book and why he was hiding it? … I asked him about it and when I looked…food which is in the book was the food which was filled in the fridge. I thought this guy has HIV and I asked him about it. He said “No” it was just a book he got from the clinic and that he was not hiding it. He did not know how it got under the bed. I kept quiet and one day I went to his house for cleaning. I cleaned for him. It was during then when I was about to leave he came inside and grabbed me. I tried to say no. I said “NO I did not want” because I was confused about the book and he was sick, coughing. He used to cough. He grabbed me and he had sex with me. From that time I knew that if this person is HIV positive then I am positive too. Some months went by then he died and I knew exactly that he died of AIDS. From that time I told myself that if I can go and check surely they will tell me that I am HIV positive…. I continued doing this thing [sex work] and I got infected so many times because I have been doing it with truck drivers without a condom.

-Nonhlanha (32, rural)

### Social Support

Both sex work and HIV are highly stigmatized in Swazi society. Informants tended to deal with these stigmas separately and were often more willing to disclose their HIV status than their occupation. However, living with HIV was often associated with promiscuity and so it was difficult for informants to totally disentangle the two identities. Women reported mixed strategies and varied responses from their friends and family in response to disclosure of either their HIV status or their engagement in sex work. Sex work was rarely disclosed to social network members who were not already part of the sex worker community. Some informants reported receiving financial assistance from other community members from time to time, while others reported widespread distrust among sex workers and fierce competition for clients that sometimes became violent.

Informants assumed that their families would react negatively or treat them poorly if their occupation was revealed. Some informants reported that their family and friends already suspected or assumed that they were engaged in sex work. Reactions ranged from tacit though disapproving acceptance of food and financial support that was funded by sex work, to outright ostracism.

Informant*: I’m afraid to tell them, even at home I haven’t told anyone. They once asked me where do I get the money. I deceived them that I have a boyfriend who gives me money. Then I’m able to help them.*

Interviewer*: Those who are asking you, they know that you’re not working?*

Informant*: It’s just that I send the money and they won’t ask me much.*

-Winile (34, peri-urban)

You see it can happen that some may neglect me. Like I said, at home some are Christians. If they can hear about this they can neglect me… It is just that now it’s my life, there is no other way I can get money.

-Nomthandazo (23, peri-urban)

Disclosure of HIV status tended to elicit better, although still mixed, reactions from an informant’s social network. Some informants described receiving both material and emotional support from family after revealing their status. Other informants reported being shamed and excluded by their families or their spouse’s families and being denied material – especially food – support.

They treat me well. They sometimes buy me things like vegetables. My aunt has a garden so she brings me some vegetables. They bring me any Swazi food they have.

-Nokubonga (31, peri-urban)

At one point [a support group member] told us … that she is not welcome at her marital home. You see. She continued to tell us that she is not given food at her marital home. We had to help her by donating some money so she may be able to buy some mealie meal [course maize flour] for her children.

-Phila (30, urban)

The same informant reported losing her job and feeling driven back to sex work as a direct result of HIV stigma:

Informant*: I used to work for one lady in a salon then one day I had gone out to the toilet she opened my bag. Because I was new in the salon I had not told her anything about my status. She would then monitor me because I think she saw the ARVs.*

Interviewer*: Did she ever see you taking them?*

Informant: *She saw them in my bag when she opened it, and she monitors my actions more, so when I get cuts then she would tell me not to touch so many things. This shows that so many people are not well informed about HIV. She monitored everything I did. She would also talk bluntly in front of the customers. Then I opted to stop working. When she asked me why I just told her I couldn’t work for her anymore I would rather stay at home and get a job I don’t know about. That’s the reason I ended up taking up the job am doing right now because no one judges you there.*

-Phila (30, urban)

In general, disclosure of HIV status tended to meet with a better reception than disclosure of sex work, but both met with mixed results at best and many of our informants reported fear of disclosure. Discrimination as a result of the synergistic effects between the dual stigmas of HIV and sex work was less common. Manifestations of dual stigma were most commonly reported in the health sector, although this experience was not universal.

You find them looking at you as a lesser being. There are those whom even if you can explain to them….they believe that when you are positive it is because you have loose morals. A person would say that you got HIV because you sleep around yet they don’t even know what really happened.

-Ntombifuthi (25, urban)

## Discussion

We found that sex workers who are living with HIV in Swaziland are frequently involved in a cycle of food insecurity, HIV risk, and social marginalization. Sex work is used to address food insecurity for women and their families. Many of our informants attributed their HIV infection to sex work and identified hunger as a motivation to engage in sex without a condom, increasing the risk of HIV and STI acquisition for themselves and onward HIV transmission to their clients. While sex workers were advised by local health workers to use “healthy foods” to manage their HIV and to ensure maximum efficacy of ART, their stigmatized identities of PLHIV and sex worker created a reduced ability to rely on social networks for food and monetary resources, and an increased reliance on sex work to meet these needs. While hunger was not a direct cause of ART non-adherence in this sample, informants did express anxiety about the effects of taking ART on an empty stomach and on the effects hunger would have on their disease progression. Social networks which our informants may have been able to draw on for material support in the past – friends and family from “back home” – were less likely to provide support because of our informants’ line of work and, at times, HIV status. This social ostracism reinforced reliance on sex work as a means to address food insecurity. While informants were more likely to disclose their HIV status than their occupation, the stigma surrounding HIV made it difficult for them to totally avoid associations with promiscuity or immorality.

As in other studies conducted with sex workers in South Africa and sub-Saharan Africa poverty and hunger are a large part of the sex work identity expressed by our informants [22,36]. Campbell suggests that emphasizing their own poverty and restricted options beyond sex work may help sex workers reclaim some social respectability and distance themselves from the stigma of the sex work identity [36]. This internalization of poverty may reinforce women’s drive to compete with one another for resources and clients, and make it difficult for sex workers to present a unified front when negotiating safer sex with clients. While the lived poverty of our informants was very real, the complex links between social support, poverty, and sex work stigma have implications for community-based or community organization style interventions in the region.

PHDP services should address the centrality of food insecurity and poverty in the lives and identities of sex workers who are living with HIV in Swaziland. Poverty and hunger were often directly equated, and hunger was the most pressing manifestation of poverty in our informants’ lives. Sex workers who are living with HIV spoke positively about the clinical advice they had received to “eat healthy foods,” but the definitions of “healthy foods” ranged from having any food available at all, to refraining from alcohol abuse or fried foods, to integrating more fruits and vegetables or more traditional Swazi foods into their diets. Future interventions intended to directly impact food insecurity would require careful formative research that addresses local understandings of food insecurity and “healthy foods” to ensure local appropriateness and desirability.

Managing their infection through a healthy diet was an important HIV coping strategy for many informants. Food prescription or supplementation programs may be the most direct way to address food insecurity for sex workers who are living with HIV. Combining food supplementation with nutritional counseling has been found to create greater impact than food supplements alone [18,37], and based on our findings this would likely be considered acceptable to this population. In Kenya, a program implemented by the AMPATH group provides food prescriptions not only to food insecure ART patients, but to their dependents [38], an important consideration given that many sex workers in Swaziland are using sex work to support their children. The six-month food prescription was coupled with a transition program to microfinance or agricultural training programs for patients and their family, coordination with the donor community, and investment in the local agricultural economy [38]. While the direct provision of food or branded supplements may lead to unintentional disclosure of PLHIV’s status, the AMPATH group’s efforts to combine food prescriptions, micro-enterprise, and dialogue with community members and international donors holds potential for an empowering, community based approach to PHDP sex work programming.

Many sex worker interventions tend to focus on individual level strategies that are specific to sex work – including peer education, condom promotion, or STI treatment – to decrease HIV transmission from sex worker to client or client to sex worker [39], and neglect the need to reduce structural barriers that may limit the accessibility of testing, treatment, and adherence services which are targeted to the general population. These barriers include criminalization, stigma, and cost [40], as well as the subordinate social and legal status of women in Swaziland and restricted access to education. These were all salient factors in our informants’ lives. Given the high prevalence of HIV in both the general and sex worker population in Swaziland, programs designed to improve access for sex workers who are living with HIV to testing, treatment, and adherence support are important to reduce individual viral load and onward transmission of HIV [3,29]. This type of structural intervention is important for stigmatized populations such as sex workers, because they experience barriers to care beyond those normally experienced by PLHIV [40]. Additionally, approaches that focus solely on individual level interventions amongst sex workers can create the perception of sex work as a risk vector [40]. This may inadvertently potentiate, rather than reduce, stigma and marginalization. The potential for escalation of stigma is important, as stigma and decreased social support played a marked role in increasing food insecurity and reliance on sex work amongst our informants.

Community empowerment interventions have shown positive results for HIV related outcomes, including reduced HIV prevalence and increased condom use [41]. This type of intervention works to enhance sex workers’ empowerment as a community, to address the poverty, violence, and stigma that heighten sex workers’ vulnerability to HIV, and prioritizes intervention aims that are set by the sex work community themselves [41,42]. This approach has been successfully implemented in India, Brazil, and the Dominican Republic, but to date there have been no assessments of community empowerment interventions with sex workers in southern Africa, nor have any of these interventions addressed the specific role of food security and vulnerability to HIV [43-48]. Our own findings and other qualitative work in the region suggests that working to build solidarity and collective action among sex workers may counter some of the stigma and violence experienced by sex workers who are living with HIV, and may also improve the feasibility of income generating projects [26,49]. Community empowerment projects must take into account the fact no community – sex work or otherwise – is monolithic. For any program to be successful, competition for resources between sex workers, as well as local politics and history, must be taken into account. However, this approach would allow sex workers with HIV to set their own intervention priorities, such as food security, and to establish for themselves how they would like to define and address the problem.

In the long term, efforts at the national level to improve women’s social, legal, and economic standing – including improved access to education and employment – are needed to broaden women’s economic options and to reduce the vulnerability of female sex workers [42]. In the shorter term, economic interventions such as microfinance and micro-enterprise projects can create an additional income source for sex workers and provide a buffer against financial crises [42,50]. In India, a micro-enterprise program was found to reduce HIV risk behaviors and provide a feasible and acceptable source of secondary income in a cohort of sex workers [51]. An evaluation of a program in Cambodia found that job training in hotels was acceptable to a group of former sex workers, and the authors emphasized the need to engage employers, support networks, sexual partners, and sex workers themselves for maximum impact [52]. Odek et al. showed that a micro-finance program in Kenya, facilitated by partner organizations that had long-standing relationships with the sex work community, resulted in decreased partner numbers and increased condom use amongst regular partners. Approximately half of the women enrolled in the project opted to suspend sex work after two years of participation in the microfinance program [53]. However, morbidity amongst participants resulted in a significantly lower loan return rate (65%) than is usually observed in successful microcredit programs. In sub-Saharan Africa, cash transfer interventions have also recently shown promise in reducing HIV risk behavior, possibly because they reduce the need to rely on sexual partners for financial security [54]. While we are aware of no cash transfer or other social safety net programs in sub-Saharan Africa that targets sex workers who are living with HIV, a qualitative study of a cash transfer program in Malawi evaluated the effects of the intervention on enrolled PLHIV. Informants reported that the cash transfers improved their individual and household food security, and their ability to access healthcare and ART. There was also a suggestion of decreased social marginalization and an increased ability to take out small loans from community members [55]. In South Africa, national child social grants given to caregivers of children under the age of 18 have been found to increase both household nutritional status and the ability of parents to engage in the broader workforce [56]. For sex workers who are living with HIV, micro-credit, micro-enterprise, and conditional cash transfer or social safety net programs all have promise, especially given the fact that many of the women we interviewed had entered sex work to provide for their children. These types of interventions would likely be most successful if they are designed in partnership with sex workers, consider sex worker’s romantic, social, and familial networks, and include components that focus on the continuing physical and mental health of participants.

## Conclusions

Informants described a cycle of hunger, HIV, and sex work, in which food insecurity frequently prompted sex work. Sex work, in turn, generated heightened risk of acquiring HIV, which our informants attempted to manage through eating either healthy foods or any food at all, in order to manage their infection and prevent side effects from their HIV medication. This increased dependence on food created an increased dependence on income generated from sex work. Moreover, the dual identity of sex worker and PLHIV created the potential for ostracism from social networks and a decreased ability to rely on family for material assistance. Based on the complex nature of this cycle, interventions that seek to provide PHDP services for sex workers living with HIV must address both upstream socio-economic factors and individual risk behaviors, including the roles of poverty and food insecurity in creating vulnerability. In Swaziland, these upstream risk factors include a national recession, high unemployment, ongoing drought, gender inequality, and other factors beyond the control of individual sex workers. PHDP services and interventions for SW which address the pathways through which food insecurity generates vulnerability to HIV and social marginalization, build sex workers collective efficacy to mobilize, consider individual and family level poverty alleviation, and address social and policy level changes are likely to have the greatest success.

## Competing interests

The authors declare that they have no competing interests.

## Authors’ contributions

RFM was the study coordinator, led data analysis and interpretation, and the writing of this article. ZM led the study design and implementation in Swaziland. DA assisted with data analysis and interpretation. CK and SB designed the study, provided technical support during its implementation, and contributed to data analysis and interpretation. All authors contributed to the study design, interpretation, and drafting of the article. All authors read and approved the final manuscript.

## Pre-publication history

The pre-publication history for this paper can be accessed here:

http://www.biomedcentral.com/1471-2458/14/79/prepub
